# Address to the Students of Harvard Veterinary School

**Published:** 1896-11

**Authors:** William F. Whitney

**Affiliations:** Boston


THE JOURNAL
OF
COMPARATIVE MEDICINE AND
VETERINARY ARCHIVES.

VOL. XVII. *NOVEMBER*, 1896. No. 11.

### ADDRESS TO THE STUDENTS OF HARVARD VETERINARY SCHOOL.

BY WILLIAM F. WHITNEY, M.D.,
BOSTON.

GENTLEMEN: It is my pleasant duty to welcome you to the veterinary school this year, and with this welcome it will be my privilege to say a few words that have no exact place in any of the regular lecture courses, as they are of such general application.

The profession which you are entering upon is one that is fast reaching the place it deserves. Old prejudices are being wiped away, and the full value of the study of comparative medicine in its broadest sense is being better and better appreciated. Without the domestic animals life would be restricted to a degree that is hard to realize, and we should in many ways be brought back to savagery. On this account their health and well-being are indispensable to us. It is in your care that their health will be intrusted, and not only theirs, but also ultimately that of the human race will be in your charge. As you know, many of the diseases of animals are directly communicable to man; such, for instance, as tuberculosis, glanders, anthrax, and rabies. And it will be your duty to be on guard against the first appearance of any such scourge, both for the sake of the animals and that of the human race. From this it is evident that the veterinarian must stand as the guardian of the public health, as it is to him that the first cases of these pestilences will be brought, and it is the early recognition and rapid extermination of the affected animals that will prevent the spread of the plague. We know with how little water a huge conflagration can be quenched in the beginning; and the truth of the old adage of the value of the ounce of prevention has increased in modern times to double that of 16 to 1.

On the other hand, there has been discovered the protective action of some diseases of animals on the human system, the one most favorably known being vaccinia against smallpox, and the antidiphtheritic serum prepared from horses' blood. The question of how these act is one of the most interesting problems of modern science, and is taxing the minds of the best thinkers to explain. This is no place to go into differences of their opinions, for as yet they are not absolutely settled. But it is simply instanced to show what interesting problems will be presented in the course of your studies, and in the answering of which animals alone can be used.

With this decade has come the general acceptance of the bacterial origin of disease, and antiseptic, or more properly aseptic, surgery has completely revolutionized this line of treatment. Now abscesses and septic processes following upon surgical interference are not looked upon as accidents, but as due to criminal neglect on the part of the operator, from the imperfect sterilization of his instruments and person. It is true that animals are not as susceptible as man to some of the common pus-producing organisms, and so blunders on the part of the operator are not punished quite so severely by nature as in human practice, but still there are a great many to which they quickly react. And the lack of cleanliness and aseptic precautions is as much to be reprehended in the veterinary surgeon as in any other class of practitioners. Your motto should be that you cannot wash your hands too often; and although we all know that cleanliness costs money, still it is the cheapest investment that a medical man can make. The simple laboratory experiment of the difference in the number of colonies of bacteria of a culture taken before and after sterilizing the hands is one of the best object lessons in this regard that anyone can have.

You see that the study upon which you have entered is a most liberalizing one; for it not only gives a scope to the highest faculties of the mind, but also arouses the best and noblest human qualities, viz., courage and unselfishness. To those who prefer the life of the student it offers opportunities for life-long work, not accompanied, perhaps, by the pecuniary emolument that will fall to the lot of those to whom the active and applied side of the profession is more alluring. It is well for the profession that both classes of men exist, for it is the latter that tests the work of the former, and only that which is of real intrinsic worth can survive his touch.

In one branch of the profession alone must you be prepared for disappointment, and that is in the knowledge it gives of *curing* a disease when once it has obtained a foothold. You will learn much of the way in which to alleviate suffering and aid nature in eliminating waste material. But in most cases you will practically have to stand aside and watch the disease run its course, helping judiciously where you can, and consoling yourself with the thought that it is the wisest physician who does the least harm by his treatment.

I would not for a moment, however, not have you learn all that you can on the subject of therapeutics, and apply it as faithfully as possible. But when you find that a favorable result follows the administration of a drug, be careful to prove that it can be due to this one alone, and would not have taken place just the same if the patient had been untreated, or another had been used. An honest, scientific skepticism should be the constant accompaniment of the interpretation of your results. I do not say but that the future has a great deal in store for us in this regard, and I trust it may be the good fortune of some of you to find it out; but the history of medicine thus far teaches that our knowledge of the diagnosis and prevention of disease far surpasses that of its treatment. There are signs in the air, but these point rather to the antagonistic action of the products of living (bacterial) action than that of inorganic or vegetable substances. You are to be congratulated upon the opportunity which many of you will have to see a great deal of what is doubt now settled one way or the other. It cannot for a moment be supposed but that there are as great discoveries reserved for the future as the past, and that other causes of disease besides the bacteria may be isolated. If some experiments on rabies, which have recently been conducted in the laboratory of the Cattle Commission, are confirmed, they will show the presence of a purely chemical ferment capable of producing that disease after a long period of apparent inactivity.

To you, gentlemen, who are just entering, it will be well to give a brief outline of the course you are to pursue. It is progressively graded, beginning with the fundamental studies of anatomy, physiology, chemistry, and botany, then through pathology, medical chemistry, theory and practice of medicine and surgery, into their practical application of diagnosis, prognosis, and treatment in the clinical departments.

With those of the first year you are naturally the most interested. And here let me impress upon you how important they are, since they form the groundwork of all your subsequent studies. You may have heard from others that they are dry and uninteresting, and that they are not practical. Do not believe it for an instant. There should not be a dull or stupid moment in them. They are the keynote to the whole, and it is the thorough mastery of these that will give you a command in your profession, and which at once raises the scientific above the empirical practitioner. Do not go at your studies as if they were only to be gotten through for some one else's benefit. You must realize that with your entrance into the professional school you have left behind you the lessons that are to be learned for a taskmaster, and that the subjects are to be studied for their own sake and what is in them. And that failure to master them is only your own loss, and any deception by which the worse is made to appear the better reason only recoils upon you. Look for the beautiful in all you do, and even let the odors of the dissecting-room be forgotten in the interest aroused by unravelling the intricate arrangements of the muscles you are laying bare. Remember the Eastern sage who found beauty in the ear of the mangled cur lying in the street.

Another point which may appear an elementary one, but to which you should pay the strictest attention, is the use of the English language. Commence at once to take notes of your work, and in them try to express yourself clearly. Language was not given to conceal, but to express our thoughts, and many men of good ideas are handicapped through life by the inability to convey them clearly to others. We have no right to ask others to read what we present to them in an illogical and ill-digested way. Anyone of you may be fortunate enough to discover something of value to the world, and you should always have a vehicle at your command by which it can be carried to the most distant parts of the earth. And that is a proper mastery both in writing and speaking of your own tongue. The ignorance upon this subject has been forced upon me year after year in reading the examination-books, which of course are written hurriedly, but still, with all allowance, the crudeness of expression is really appalling. It is with this in view that quiz classes and written conferences by the students are of great value. Life is one long quiz, and the sooner you become prepared to answer questions, the easier it will be to reply to those that will be showered upon you the moment you commence the battle of life. And the school is the place where you can prepare for this, without exposing yourself to ridicule. Never be afraid to acknowledge your ignorance, and do not allow that you understand a point or subject until you really do. The instructor may be at fault in his presentation of it, and therein lies the great advantage of many teachers going over the same ground from different points of view, from some one of which it will probably become clear to you.

A faculty which you should begin to cultivate at once is that of observation, for it is upon this more than anything else that your success depends. Your patients cannot express their feelings in words, and so cannot aid you in locating their pains, and thus help you in your diagnosis. You must learn all of this from observation of them. But it is not alone to this that it is applicable; through your whole course seeing for yourself is what you are expected to do, and one fact properly brought to light from its entangling surroundings by yourself is worth fifty that are pointed out to you by someone else. Progress would be at a standstill if nothing new was discovered. And discovery means the observation of something that has hitherto been overlooked.

The trite remark that "I never saw that before," as a picturesque bit of landscape is pointed out to us by an artist friend in our daily walk, shows us that we all have eyes but see not, and will convey to you what I mean by cultivating observation. But it is not only seeing, but applying what is seen, that gives the highest class of minds; and when the application is in new and untrodden paths, then come those flashes of genius that illuminate the world. Now, although we cannot all hope to attain to this plane, still the cultivation of observation and the application of it are the stepping-stone, and if we have the right combination in ourselves the highest prizes are within our reach.

To those who are about to leave the school much that has been said is already well known perhaps, but still we are none the less students after our degrees have been received than before. The only difference is, that the problems which are presented to us are of all sorts and degrees of complexity, and not arranged in the graded way of the regular curriculum. Let the same high aim be present in your work by yourself as when you were in the class with others. Do not degenerate into a routine practitioner, who sees nothing in his profession but a means of earning a livelihood. While I would not for a moment wish to depreciate the value of your services, and that they should not receive a due pecuniary reward, still there is something else which will bring you more true happiness and mental pleasure. Be on the lookout for something new all the time, and try to contribute something to the advancement of your profession, in return for the accumulated knowledge of your predecessors which has been so freely given to you. But while urging you to do this, I wish to warn you against rushing too quickly into print with premature conclusions. Put your discoveries away for a time, and after a little, if they still shine with equal lustre under the touchstone of your subsequent experience, they can be given to the world. Begin at once to keep records of your cases taken on the spot and do not trust to your memory; for the impression quickly fades under the rapid succession of new facts, and it is difficult to find time later to jot down what are even the striking features of a case. A few words written at the time are worth more for future use than reams done later from memory. In this way you will soon have a fund of information of your own accumulation, to which you will refer with ever-increasing interest and profit.

You can also help the profession by aiding the school, for besides the instruction there is another side to the school and hospital—viz., that of investigation. And this it is impossible for the practitioner to carry on alone; it requires a laboratory and other equipments which are out of the reach of the individual. They will only too gladly be placed at the disposal of any of you who have the inclination and the fitness for that peculiar kind of work. So let your influence be exerted wherever it can to induce students to come or friends to contribute to the institution, which has for its final aim the alleviation of suffering and advancement of knowledge. And it is in this spirit that the alumni association should be fostered, not in any clannish or narrow one, but with a view to keeping up your old friendships and sympathy for your alma mater, bringing to her as a repository all that is new and best, either in knowledge or means, from year to year.

In closing, let me ask you always to bear in mind that it is the men who give the dignity to the profession, and not the profession to the men, and to so bear yourselves in your relation to your clients that it will always be considered an honor to be enrolled in the profession of which you are members. Never let a taint of charlatanism or chicanery be associated with your practice, and let all you do be of such a character that full light can be turned on it at any time. And bear always in mind what has so well been said by the immortal student of human nature:

> "To thine own self be true,
> And it must follow as the night the day
> Thou canst not be false to any man."

